# Histone lactylation-derived TET2 enhanced Arg1-mediated MDSC immunosuppression

**DOI:** 10.3389/fimmu.2025.1677780

**Published:** 2026-01-16

**Authors:** Wenxin Da, Yao Dai, Bo Shen, Yan Zhang, Pengtao Bao, Wei Zhu, Deqiang Wang, Shengjun Wang, Jie Ma

**Affiliations:** 1 Department of Immunology, School of Medicine, Jiangsu University, Zhenjiang, Jiangsu, China; 2 Jiangsu Key Laboratory of Laboratory Medicine, Department of Laboratory Medicine, School of Medicine, Jiangsu University, Zhenjiang, Jiangsu, China; 3 The Affiliated Cancer Hospital of Nanjing Medical University, Jiangsu Cancer Hospital & Jiangsu Institute of Cancer Research, Nanjing, Jiangsu, China; 4 College of Pulmonary & Critical Care Medicine, 8th Medical Center, Chinese People’s Liberation Army (PLA) General Hospital, Beijing, China; 5 Department of Oncology, Institute of Digestive Diseases, The Affiliated Hospital of Jiangsu University, Zhenjiang, Jiangsu, China

**Keywords:** Arg1, lactylation, MDSCs, STAT3, TET2

## Abstract

In the tumor microenvironment (TME), tumor cells secrete a large amounts of lactate due to the “Warburg effect”, which plays a significant role in regulating gene transcription. Recently, the role of lactate in gene transcription has been increasingly understood. Myeloid-derived suppressor cells (MDSCs) are inhibitory cells of bone marrow origin that possess marked abilities to suppress immune cell responses. Within the TME, MDSCs inhibit T cell-mediated specific anti-tumor immunity, as well as non-specific anti-tumor immunity mediated by NK cells and macrophages, by expressing high levels of Arg1, iNOS, and ROS.

**Methods:**

This study used the Lewis lung carcinoma cell line to establish a lung cancer xenograft model; MDSCs were isolated from the spleens of these mice for subsequent experiments. Protein expression was analyzed by Western blotting, mRNA expression by qRT-PCR, protein-DNA interactions by ChIP-qPCR, and DNA methylation by MSP-qPCR.

**Result:**

This research shows that histone lactylation enhances the immunosuppressive function of MDSCs. Mechanistically, lactate-induced histone lactylation upregulates TET2, which, using STAT3 as a bridge, modulates ARG1 promoter methylation to upregulate its expression and ultimately enhance the immunosuppressive function of MDSCs.

**Conclusion:**

This research reveals that the histone lactylation-mediated alteration of TET2 presents a novel therapeutic target for cancer treatment.

## Introduction

Cancer is one of the leading causes of death worldwide, as identified by the WHO, causing tens of thousands of deaths annually (1). The tumor microenvironment (TME) is a highly structured ecosystem intimately involved in tumor development, comprising surrounding blood vessels, immune cells, various signaling molecules, and the extracellular matrix (2). Immune cells within the TME play crucial roles, including immune-suppressive cells such as regulatory T cells (Treg) and tumor-associated macrophages (TAM), as well as immune-activating cells such as CD8^+^T cells and natural killer (NK) cells (3). By regulating the quantity and activity of immune cells, the TME can counteract the immune response to the tumor. The complex nature of TME results in the inhibition of the immune response during tumor progression, with macrophages, dendritic cells (DCs), neutrophils, myeloid-derived suppressor cells (MDSCs), NK cells, and innate lymphoid cells acting as “accomplices” in tumor initiation and development (3). Within the TME, there are abundant immune cells, among which MDSCs are a heterogeneous group of bone marrow cells with immunosuppressive characteristics, originating from myeloid progenitor cells and immature myeloid cells (4). MDSCs are distributed in various organs such as bone marrow and spleen, where they maintain their immunosuppressive function and regulate immune responses (5). MDSCs in the TME collectively promote tumor development through various means and contribute to the formation of an immune-suppressive environment, which in turn influences the biology and function of MDSCs. Firstly, MDSCs express negative immune checkpoint molecules to block T cell function. Numerous studies have shown that tumor-infiltrating MDSCs often exhibit higher levels of PD-L1 expression, largely associated with their hypoxic microenvironment (6, 7). Secondly, arginase 1 (Arg1), reactive oxygen species (ROS), and nitric oxide (NO) collectively deplete T cell nutrients and impair T cell function, resulting in an immunosuppressive microenvironment that hinders immune responses (8). Furthermore, within the complex environment of TME, various metabolites and cellular products have been shown to enhance the immunosuppressive function of MDSCs. In a murine melanoma research model, it has been demonstrated that tumor cells, MDSCs, and Treg can secrete extracellular adenosine to suppress T cell function (9). Additionally, it has been found that certain metabolic byproducts in TME, such as lactate, cholesterol, and amino acids, can also regulate the function of MDSCs, promoting tumor progression (10).

Due to the “Warburg effect,” a large amount of lactate is produced in TME, leading to an acidic pH. This acidic condition favors processes such as tumor growth, metastasis, and angiogenesis (11). In recent years, there has been increasing understanding of lactate. In 2019, Professor Yingming Zhao and his team first proposed the concept of lysine lactylation on histones, a novel post-translational modification of proteins (12). Through high-performance liquid chromatography-tandem mass spectrometry analysis, researchers observed a 72.021 kDa mass shift on the lysine residues of the core histones from trypsin-digested human MCF-7 cells. Subsequently, with the aid of the isotope-labeled L-lactate, this was confirmed to be histone lactylation derived from L-lactate. Consequently, more studies have begun to explore the function and mechanism of lactylation modification.

The tumor microenvironment TME, characterized by high lactate levels and hypoxia (as shown by Professor Zhao’s work), intrinsically promotes lactylation. This modification plays a multifaceted role in oncogenesis. For instance, in ocular melanoma, histone lactylation activates the reader protein YTHDF2, revealing a novel therapeutic target (13). Furthermore, lactylation is pivotal in immune evasion. As demonstrated by Jia et al., lactate in the TME upregulates METTL3 in tumor-infiltrating myeloid cells via H3K18la. This is enhanced by lactylation at two sites within METTL3’s zinc finger domain, and the subsequent lactylation-driven, METTL3-mediated RNA m6A modification is crucial for amplifying TIM immunosuppressive function (14). Collectively, this evidence underscores the significant role of lactylation in tumor initiation and progression.

Lactylation is a novel histone modification regulated by specific enzymes known as “writers,” “erasers,” and “readers,” which respectively mediate the addition, removal, and recognition of this post-translational modification. The histone acetyltransferase p300 was later identified by Zhang et al. as a lactylation writer, confirming its role in catalyzing histone lysine lactylation. Similarly, the existence of lactylation erasers was subsequently discovered. Following key insights into lactylation by Professor Zhao’s team, Moreno-Yruela et al. demonstrated that two deacetylase families HDAC1–3 and SIRT1–3 also function as delactylases (15). Thus, p300, HDAC1-3, and SIRT1–3 have been established as core regulators of histone lactylation.

The Ten-Eleven Translocation (TET) proteins catalyze the oxidation of 5-methylcytosine (5mC) to generate 5-hydroxymethylcytosine (5hmC), thereby participating in the regulation of DNA methylation stability. They play crucial roles in various physiological and pathological processes (16, 17). TET1, TET2, and TET3 are three members of the TET family, each with unique functions in regulating 5hmC. TET1 consistently controls 5hmC at transcription start sites, TET2 is associated with gene body 5hmC, while TET3 has less characterized demethylating activity and has been less studied (18). In recent years, TET2, as a member of the TET family, has been widely recognized as a crucial regulator of normal hematopoiesis, particularly in bone marrow development. Its dysregulation can lead to the development of various hematologic malignancies, and its role in solid tumors should not be underestimated (19–21). Furthermore, TET2 plays a crucial role in immune homeostasis by promoting DNA demethylation or through mechanisms independent of its enzymatic activity. Evidence suggests that tumor-associated macrophages with reduced TET2 expression exhibit altered immune activity, including increased expression of inflammatory cytokines and decreased Arg1 expression, thereby enhancing anti-tumor T cell responses (22).

In this study, we identified, for the first time, that increased lactate levels enhance the immunosuppressive function of MDSCs. Further experiments revealed that lactate upregulate TET2 expression, and inhibition of lactate uptake leads to decreased TET2 expression, accompanied by a reduction in the immunosuppressive function of MDSCs. Additionally, we demonstrated that TET2 mediates the demethylation of the Arg1 promoter, thereby impacting Arg1 expression and the immunosuppressive function of MDSCs. Therefore, our data reveal a novel mechanism in MDSCs: lactate upregulates TET2 expression to enhance MDSC function, highlighting the important role of lactate and its lactylation modifications in tumor development.

## Material and methods

### Cell lines and cell cultures

The lewis lung carcinoma cell line was purchased from Shanghai sangon biotech. (Sangon, China). Lewis cells were cultured in DMEM (Servicebio, China) complete medium supplemented with antibiotics (penicillin and streptomycin) (Enzo, USA) and 10% fetal bovine serum (FBS) (Gibco, USA).

### Establishment of lung cancer model

Twelve male C57BL/6 mice (6–8 weeks old, weighing 20-22g) were obtained from the Jiangsu University Animal Center (certification number A202401120085). All animal experiments were approved by the Institutional Animal Care and Use Committee of Jiangsu University. The mice were housed in groups of six per cage in a controlled environment with a 12-hour light/dark cycle, and provided ad libitum access to water and food.

To establish a Lewis cell xenograft tumor model, Lewis cells were cultured to 80-90% confluency, trypsinized carefully, and injected subcutaneously into mice. For the animal experiments, a total of 20 mice were randomly divided into two groups of ten: tumor-bearing group and control group. The inclusion criterion for the mice was a weight between 20–22 grams. The exclusion criterion was based on the failure to establish the tumor-bearing model. Specifically, if mice did not show expected tumor growth or exhibited significant health issues after implantation, they were excluded. A total of 22 mice were used, and 2 were excluded due to unsuccessful tumor implantation. Tumor growth and animal health were monitored daily. Appropriate animals were selected, the environment was controlled, procedures were standardized, and data were recorded to ensure accurate results.

### Immunomagnetic bead separation of MDSCs

Without pre-charging the chamber, place the mice in the chamber and introduce 100% CO2 at a fill rate of 30-70% displacement of the chamber volume per minute with CO2, added to the existing air in the chamber. Once the animals gradually lose consciousness, the carbon dioxide concentration was increased to 100%. Unconsciousness was confirmed by the absence of toe pinch reflex, loss of muscle tone, and no corneal reflex. Ventilation continued for 2 minutes to confirm death. To isolate MDSCs from mouse spleen, the spleen was disrupted through a 70 μm cell strainer (Biosharp, China). Red blood cells were lysed using a lysis buffer, and the lysis reaction was quenched with 1640 (Servicebio, China) complete medium containing 10% FBS. The cells were centrifuged and the supernatant was discarded. The cells were washed twice with PBS (Servicebio, China) containing 0.1% BSA (Biosharp, China). Cells were resuspended in PBE buffer and incubated with CD11b-biotin magnetic beads (Biolegend, USA) at 4 °C. The cells were washed twice with PBS buffer and centrifuged. Cells were resuspended in PBS buffer and added dropwise to a magnetic separation column (MACS, USA) pre-equilibrated with PBS buffer. Finally, MDSCs were eluted from the column using PBS buffer. The final suspension volume was approximately 10 mL.

### Flow cytometry for MDSC purity assessment

PE-Gr-1 antibody and PE-Cy5-CD11b antibody (Biolegend, USA) were added to the isolated MDSCs (1.5×10^5^ cells), incubated at 4 °C for 30 minutes, washed with 1 mL PBS, centrifuged, the supernatant discarded, and the MDSCs resuspended in 200 μL PBS. Flow cytometry (FCM) was then used to detect the purity of MDSCs.

### qRT-PCR detection of mRNA levels in MDSCs

RNA was extracted from MDSCs using TRIzolTM reagent (Thermo Fisher Scientific, USA), according to the manufacturer’s instructions. RNA concentrations were measured using a NanoDrop One (Thermo Fisher Scientific, USA). RNA (1000 ng) was reverse transcribed using a Reverse Transcription Kit (Vazyme, China). Quantitative real-time PCR (qPCR) was performed using the primers listed below and SYBR reagents (Bio-rad, USA). The primers used are:

β-Actin: FORWARD: 5’- TTGTGATGGACTCCGGAGAC -3’

REVERSE: 5’- TGATGTCACGCACGATTTCC -3’

TET2: FORWARD:5’-CAGAGGCTGCCCCTGCTAAAC-3’

REVERSE:5’-CTTCTGTTTACTTTGGCCACGTGGG-3’

Arg1: FORWARD: 5’-GGACCTGGCCTTTGTTGATG-3’

REVERSE:5’- CCAGAGATGCTTCCAACTGC-3’

### Quantitative methylation specific PCR (MSP-qPCR)

Genomic DNA was extracted and subjected to bisulfite treatment, converting unmethylated cytosines to uracil. Specific primers targeting methylated and unmethylated DNA sequences were designed, and qPCR reactions were conducted. PCR products were detected using fluorescent dyes, and methylation levels were calculated using the ^△△^Ct method. Methylation levels of each sample were compared to the control group to determine methylation differences and draw conclusions about gene expression, disease occurrence, and prognosis.

Primers for MSP-qPCR used are:

#### Arg1 methylated-specific primer

FORWARD: 5’-TTTTTTTGTATTAAATGGGTTTTTC-3’

REVERSE: 5’- AAACAATAAACATTCTACACCCG-3’

#### Arg1 unmethylated-specific primer

FORWARD: 5’-TTTTTGTATTAAATGGGTTTTTTGG-3’

REVERSE: 5’- AAACAATAAACATTCTACACCCACC-3’

### Western blot analysis

Cell and tissue extracts were prepared using RIPA lysis buffer (Thermo Fisher, USA). Briefly, after SDS-polyacrylamide gel electrophoresis, proteins were transferred onto PVDF membranes (Bio-Rad, USA). The membranes were blocked with 5% skim milk and then incubated overnight at 4°C with primary antibodies against β-Actin, TET2, p300, STAT3, p-STAT3, Pan Kla, H3K18la, H4K12la, H3, and Arg1. After washing three times with TBST, the membranes were incubated with HRP-conjugated secondary antibodies for 1h at 37°C. Following another three washes with TBST (15min each), the blots were visualized using an ECL reagent (Thermo Fisher, USA). The protein levels of TET2 (21207-1-AP, Proteintech, China), p300 (86377T, Cell Signaling Technology, USA), STAT3 (9139T, Cell Signaling Technology, USA), p-STAT3 (9145T, Cell Signaling Technology, USA), and Arg1 (82975-1-RR, Proteintech, China) were normalized to β-Actin (60008-1-Ig, Proteintech, China), while the levels of Pan Kla (PTM-1401RM, PTM Bio, China), H3K18la (PTM-1427RM, PTM Bio, China), and H4K12la (PTM-1411RM, PTM Bio, China) were normalized to histone H3.

### Chromatin immunoprecipitation assays

Chromatin immunoprecipitation (ChIP) assays were performed using the SimpleChIP^®^ Plus Enzymatic Chromatin IP Kit (Magnetic Beads) (Cell Signaling Technology, USA). Briefly, cells were fixed with formaldehyde for cross-linking, followed by lysis of membranes and fragmentation of chromatin with micrococcal nuclease. The digested chromatin was then immunoprecipitated by incubation with an anti-Flag antibody (Cell Signaling Technology, USA). Histone H3 antibody (Cell Signaling Technology, USA) and normal human IgG (Cell Signaling Technology, USA) served as positive and negative controls, respectively. Following extensive washing, the immunoprecipitated DNA was eluted from the beads and analyzed by quantitative real-time PCR (qPCR).

The primers used are:

TET2-1: FORWARD:5’-CAGAGGCTGCCCCTGCTAAACTTTAA -3’

REVERSE:5’- GGATGTTCTTTACTGGCTTGCTTCC -3’

TET2-2: FORWARD:5’- ATAAGAGAGCAGGTTGAGCAAGC -3’

REVERSE:5’- TATGTACTCACACAGGAAAGGCCAGG-3’

TET2-3: FORWARD:5’- ACACAGAGTAACAATGTGCCACA -3’

REVERSE: 5’- AGGTAATCTGGGAACCTCTTCTGT -3’

TET2-4: FORWARD: 5’- AAGTAGACTTAAGGTTGCTGTGAAC -3’

REVERSE: 5’- GAATTAAGCCAGCAGTTTCTAATGTTCA-3’

Arg1-1: FORWARD:5’-AGACAAAGGGGAAATGGAGGATT-3’

REVERSE:5’- TGAGGGACTTTGGCTTCTTGATT -3’

Arg1-2: FORWARD:5’-TGCAGAAGAATCGAAACGGA-3’

REVERSE: 5’- TTGGCACACGACAAAGACAA-3’

Arg1-3: FORWARD: 5’-AAAAAGCCAGCTGGATGACA-3’

REVERSE: 5’-ATCCCAAATGTGCACCTCTACAT-3’

### siRNA-mediated gene knockdown

siRNAs targeting the mouse TET2 gene and a negative control siRNA were synthesized by Shanghai GenePharma. The sequences for TET2-siRNA were: sense strand 5’-CAGGGAUCUACAUAGAUAUTT-3’, antisense strand 5’-AUAUCUAUGUAGAUCCCUGTT-3’.

Transfection was performed using Lipofectamine 3000 reagent. Briefly, when cells reached 60-70% confluence, 100 nM siRNA was mixed with Lipofectamine 3000 and P3000 Enhancer in Opti-MEM medium. The mixture was incubated at room temperature for 15 minutes and then added to the cells. The culture medium was replaced with fresh complete medium 6 hours post-transfection.

The knockdown efficiency of TET2 was validated at the mRNA and protein levels by qRT-PCR and Western blotting at 48 hours and 72 hours post-transfection, respectively.

### Statistical analysis

One-way ANOVA followed by the Bonferroni test was used for multiple group comparisons with Graphpad prism 8.0. The two-tailed Student’s t-test was used for comparisons between two groups. The experimental data are expressed as the mean ± SEM. Student’s t-test and ANOVA were used to determine the statistical differences. The differences at p<0.05 were considered statistically significant.

## Results

### Lactate upregulates the immunosuppressive function of MDSCs

**Figure 1:**
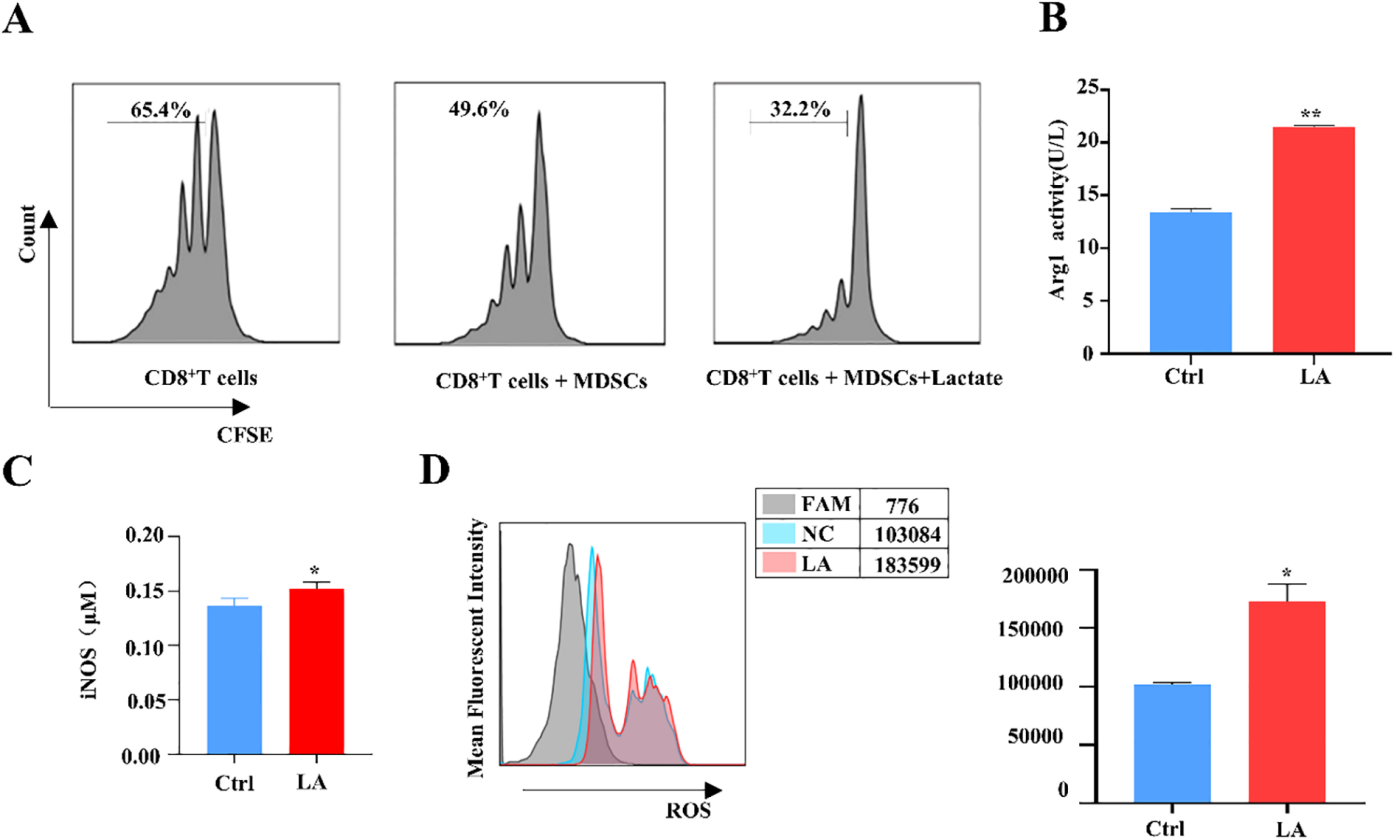
Lactate upregulates the immunosuppressive function of MDSCs. **(A)** The immunosuppressive effect of MDSCs on CD8^+^T cell proliferation before and after lactate treatment (20 mM) was assessed by flow cytometry. **(B)** The Arg1 activity of MDSCs after lactate treatment was assessed using an Arg1 activity assay kit. **(C)** The expression of iNOS in MDSCs was analyzed following lactate treatment by iNOS assay kit. **(D)** The expression of ROS in MDSCs was analyzed following lactate treatment by flow cytometry. All data are presented as mean ± SD from n = 6 independent biological replicates. Each experiment was independently repeated three times with consistent results. *p <0.05, **p<0.01.

We first established subcutaneous tumor model in mice using Lewis lung cancer cells. Following model establishment, MDSCs were isolated from the spleens of the lung cancer tumor-bearing mice using magnetic bead separation, and the purity of the MDSCs was assessed using FCM. The results indicated that the purity of isolated MDSCs was 91.7%, meeting the requirements for subsequent experiments (**Supplementary Figure S1A**). To investigate the changes in the immunosuppressive function of MDSCs before and after lactate treatment, we used FCM to assess the proliferation of CD8^+^ T cells co-cultured with MDSCs. The CD8^+^ T cells, which were isolated from the spleen with a purity of 90.8%, were co-cultured with MDSCs for this assay (**Supplementary Figure S1B**). The results demonstrated a significant enhancement of the inhibitory effect of MDSCs on CD8^+^T cells proliferation following lactate treatment, indicating enhanced immunosuppressive function (**Figure 1A**). We then used an Arg1 activity assay kit to measure Arg1 activity and found it was significantly upregulated in MDSCs after lactate treatment (**Figure 1B**). Finally, detect the expression levels of iNOS and the levels of ROS using flow cytometry. Similarly, ROS and iNOS levels were significantly upregulated (**Figures 1C, D**). These results suggest that lactate may upregulates the immunosuppressive function of MDSCs.

### Lactate upregulates TET2 expression, enhancing MDSCs function

**Figure 2:**
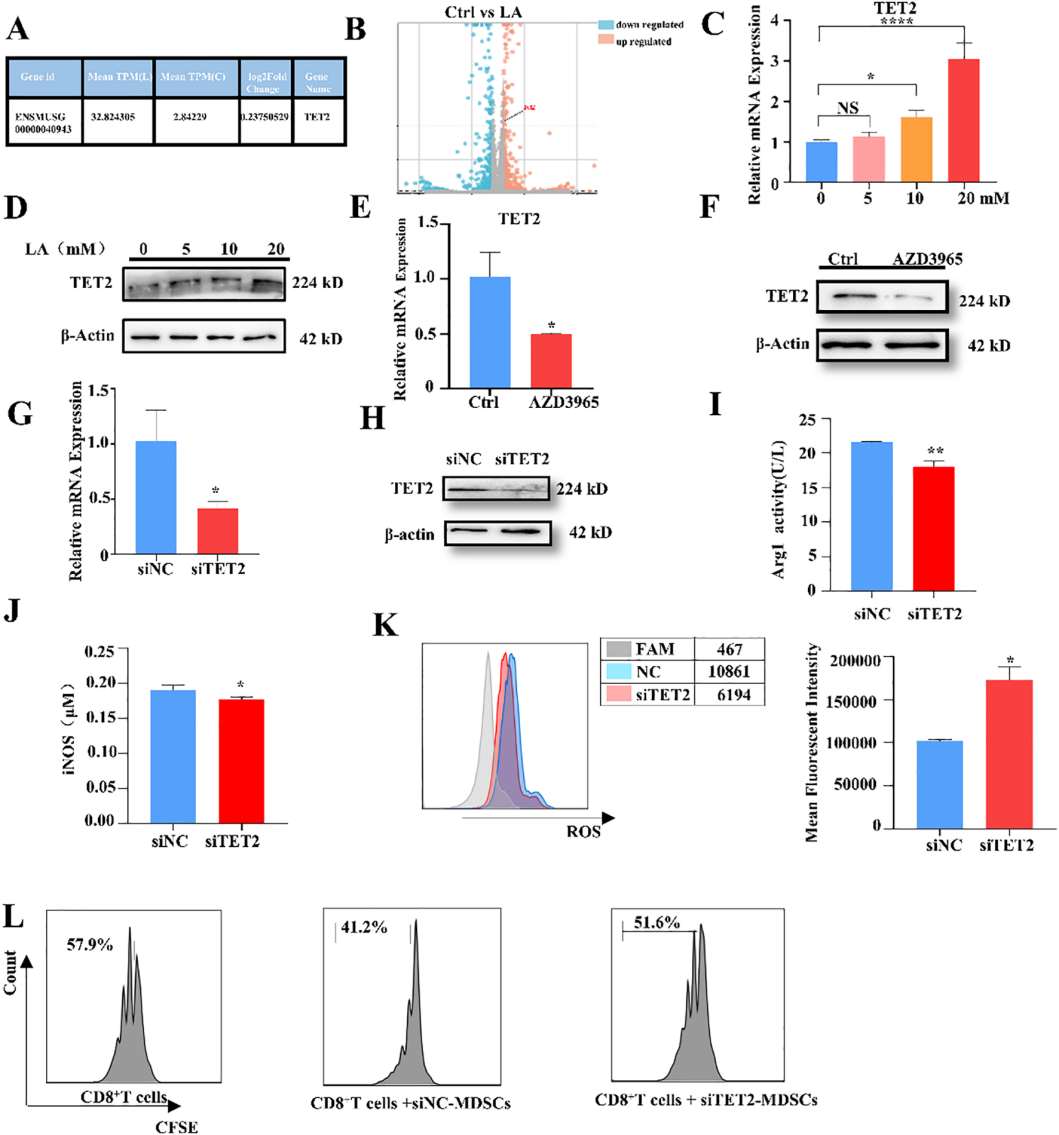
Lactate upregulates TET2 expression, resulting in enhanced MDSC function. **(A)** Lactate treatment resulted in a significant upregulation of TET2 genomic expression levels. **(B)** Volcano plot revealed altered TET2 expression patterns in MDSCs following lactate treatment. **(C)** TET2 expression levels after lactate treatment at concentrations of 0–20 mM. **(D)** TET2 expression levels in MDSCs cultured in different concentrations of lactate by western blot. **(E)** The expression levels of TET2 in MDSCs after treatment with AZD3965 by qRT-PCR. **(F)** The protein expression levels of TET2 in MDSCs by Western blot after treatment with AZD3965. **(G)** The knockdown efficiency of siRNA-TET2 by qRT-PCR. **(H)** The knockdown efficiency of siRNA-TET2 by Western blot. **(I)** The Arg1 activity of MDSCs following siRNA-TET2 was assessed using an Arg1 activity assay kit. **(J)** The expression of iNOS in MDSCs was analyzed following siRNA-TET2 by iNOS assay kit. **(K)** Flow cytometry was employed to quantify ROS production in MDSCs following siRNA-TET2. **(L)** The ability to suppress CD8^+^ T cell proliferation by flow cytometry after siRNA-TET2. All quantitative data represent the mean ± SD of n = 6 independent biological replicates. The findings were consistent across three independent experimental replicates. *p < 0.05, **p < 0.01, ****p < 0.001; NS, no significance.

To further explore the target genes involved in regulating MDSCs immunosuppressive function, whole-genome transcriptome sequencing analysis was conducted on lactate-treated MDSCs. The results showed that 236 significantly upregulated genes and 269 significantly downregulated genes after lactate treatment. Among these, TET2 expression was significantly increased, as indicated by the gene heatmap and sequencing results (**Figures 2A, B**). Treatment with lactate at varying concentrations revealed that 20 mM lactate yielded the highest levels of both TET2 mRNA and protein in MDSCs. (**Figures 2C, D**). Lactate is frequently transported into cells via monocarboxylate transporters (MCTs), predominantly MCT1 (23). Therefore, MDSCs were treated with the MCT1 inhibitor AZD3965. Inhibition of lactate uptake resulted in decreased TET2 expression (**Figures 2E, F**). These results indicate that lactate increases TET2 expression levels.

Following the elucidation of lactate’s effect on TET2, we focused on the role of TET2 in MDSCs. SiRNA was used to knockdown TET2 expression in MDSCs. qRT-PCR and Western blot confirmed the efficacy of siRNA-TET2 (**Figures 2G, H**). Subsequent results showed that the activity of Arg1, the levels of iNOS, and the levels of ROS significantly decreased after the use of siRNA-TET2 (**Figures 2I–K**). Co-culture of siRNA-TET2 transfected MDSCs with CD8^+^T cells and FCM analysis of T cell proliferation confirmed a significant downregulation of MDSCs immunosuppressive function (**Figure 2J**). These results indicate that TET2 enhances the immunosuppressive function of MDSCs. Therefore, the data demonstrate that lactate upregulates TET2 in MDSCs, thereby impacting their immune function.

### Lactate mediates histone lactylation to regulate the TET2 promoter

**Figure 3:**
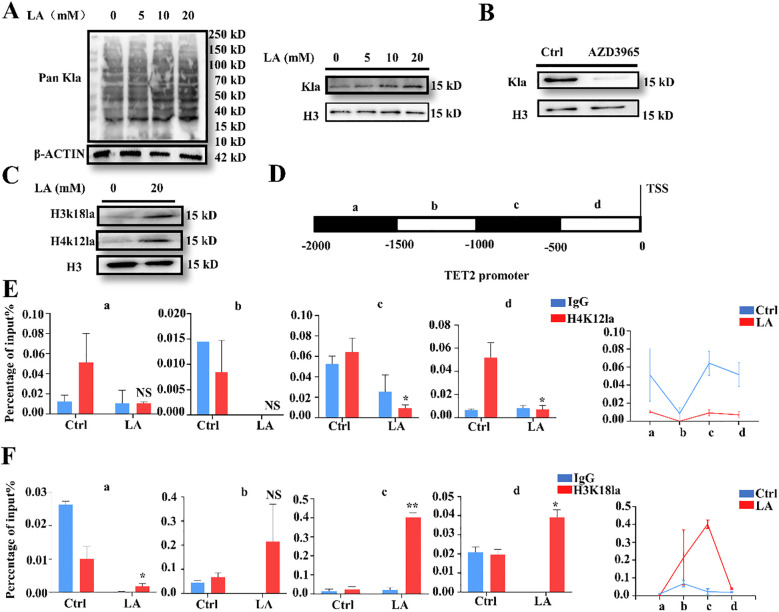
Lactate mediates histone lactylation to regulate the TET2 promoter. **(A)** The Pan-Kla levels in MDSCs after lactate treatment were assessed by Western blot. **(B)** The histone lactylation levels in MDSCs after AZD3965 treatment were investigated by Western blot. **(C)** Western blot was performed to test H3k18la and H4k12la expression in MDSCs. **(D)** Design of TET2 promoter primers. **(E)** ChIP-qPCR assay of H4K12la status in the TET2 genomic region in MDSCs. **(F)** ChIP-qPCR assay of H3K18la status in the TET2 genomic region in MDSCs. All quantitative data represent the mean ± SD of n = 6 independent biological replicates. The findings were consistent across three independent experimental replicates. *p < 0.05, **p < 0.01; NS, no significance.

The next step aimed to investigate the specific mechanism by which lactate upregulates TET2. We first explored whether lactylation levels change after lactate treatment. MDSCs were treated with different concentrations of lactate (0,5,10,20 mM). After 24 hours, Western blot detected changes in Pan-lactylation (Pan-Kla) levels. Results showed a significant increase in Pan-Kla levels, as well as specific histone lactylation levels, in MDSCs with increasing lactate concentrations (**Figure 3A**). Lactate generally enters cells via MCT-mediated transport, with MCT1 playing a predominant role (24). Therefore, MDSCs were treated with the MCT1 inhibitor AZD3965(10 nM) to inhibit exogenous lactate entry. Lactylation levels in MDSCs were significantly downregulated (**Figure 3B**).

To uncover the regulatory role of histone lactylation in TET2 gene expression, we first detected the lactylation levels of the histone H3 lysine 18 (H3K18 la) and histone H4 lysine 12 (H4K12la) by Western blot as current research on histone lactylation primarily focuses on these two sites (25–27). Levels at both sites increased significantly (**Figure 3C**). We designed primers targeting the TET2 promoter region for ChIP-qPCR analysis (**Figure 3D**). ChIP-qPCR results revealed significant enrichment of H3K18la, but not H4K12la, within the TET2 promoter region (**Figures 3E, F**). This indicated that the change in TET2 expression levels is associated with enrichment of lactylation at H3K18 on histones within its promoter.

### P300 and SIRT1 regulate histone lactylation modification to control TET2 expression levels

**Figure 4:**
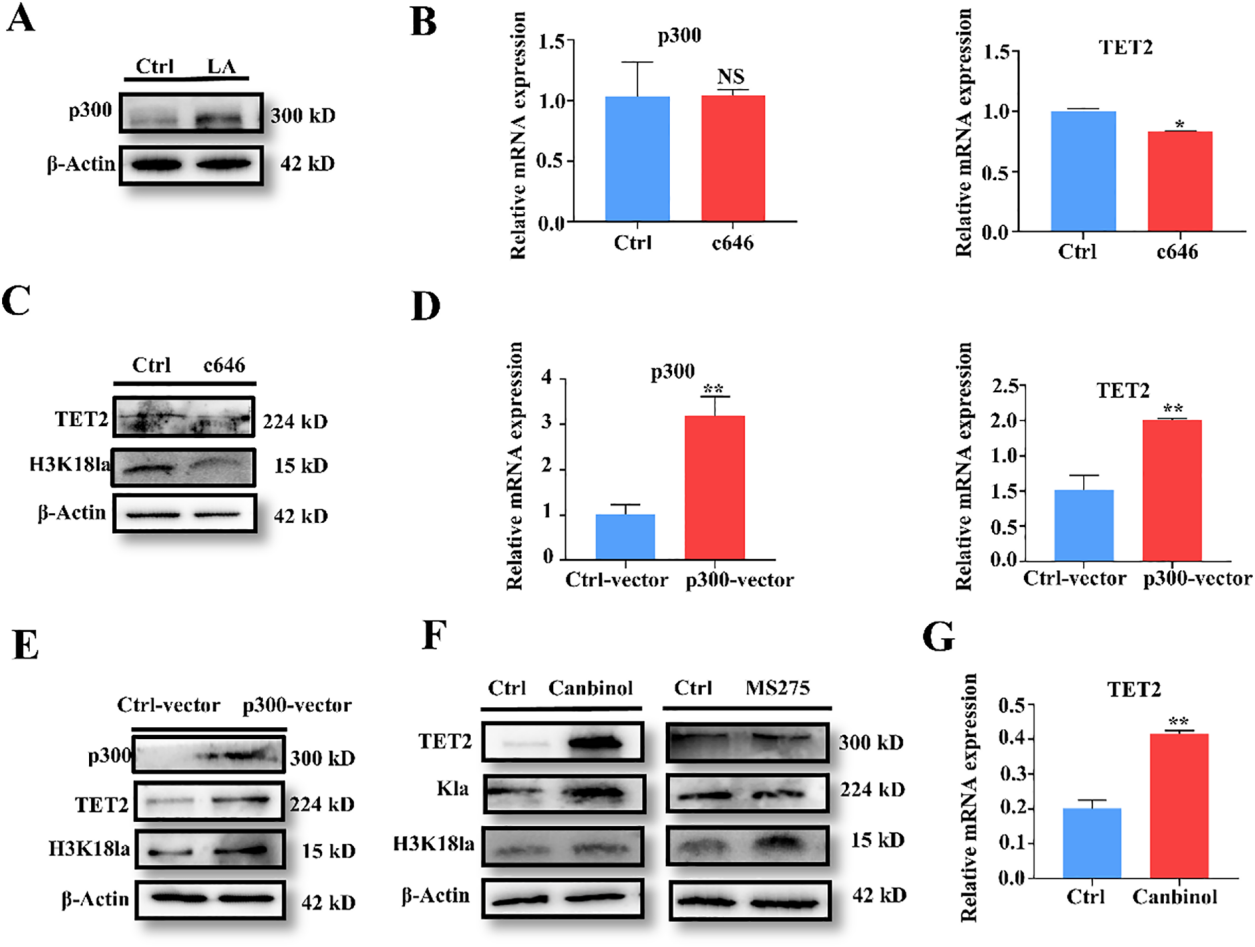
P300 and SIRT1 regulate histone lactylation modification to control the expression levels of TET2. **(A)** Western blot analysis of p300 protein expression following lactate treatment in MDSCs. **(B)** qRT-PCR showed that p300 and TET2 after c646 treatment in MDSCs. **(C)** Western blot analysis of TET2 and H3K18la protein expression following lactate treatment. **(D)** qRT-PCR showed that p300 and TET2 following p300 overexpression in MDSCs. **(E)** The effect of p300 overexpression on TET2 protein expression was investigated by western blot. **(F)** Western blot analysis showed that the expression levels of TET2 after treatment with SIRT and HDAC inhibitors. **(G)** qRT-PCR results show TET2 expression levels following Cambinol treatment. All quantitative data represent the mean ± SD of n = 6 independent biological replicates. The findings were consistent across three independent experimental replicates. *p < 0.05, **p < 0.01; NS, no significance.

Based on Professor Zhao’s research, p300 is a key protein involved in histone lactylation modification (12). We initially observed increased p300 expression in lactate-treated MDSCs by Western blot (**Figure 4A**). To investigate p300’s effect on TET2, MDSCs were treated with the p300 inhibitor C646(10 μM). QRT-PCR showed significant downregulation of TET2 expression (**Figure 4B**) (28). Protein levels of TET2 and H3K18 ac also decreased significantly following C646 treatment (**Figure 4C**). Conversely, TET2 mRNA levels significantly increased following p300 overexpression, accompanied by increased TET2 and H3K18ac protein levels (**Figures 4D, E**). suggest histone lactylation de-modification is mediated by histone deacetylases (HDAC) and sirtuins (SIRT) (15). Therefore, MDSCs were treated with the SIRT1 inhibitor Cambinol (100 mM) and the HDAC inhibitor MS275 (5 μM). Western blot showed that SIRT1 inhibition significantly increased TET2 expression and lactylation levels. In contrast, HDAC inhibition had no significant effect on either. Consistently, Cambinol treatment (a SIRT1 inhibitor) led to a significant upregulation of TET2 mRNA. (**Figures 4F, G**). This section demonstrates that SIRT1 and P300, as histone lactylation-modifying proteins, regulate TET2 expression levels.

### TET2 regulates the methylation levels of the Arg1 promoter region in MDSCs

MDSCs primarily exert immunosuppressive effects through molecules like Arg1, ROS, and iNOS (29). Results in **Figure 2** showed that knocking down TET2 using siTET2 significantly reduced Arg1 expression. Therefore, TET2 likely regulates the methylation levels in the Arg1 promoter region, influencing MDSC immunosuppressive function.

**Figure 5:**
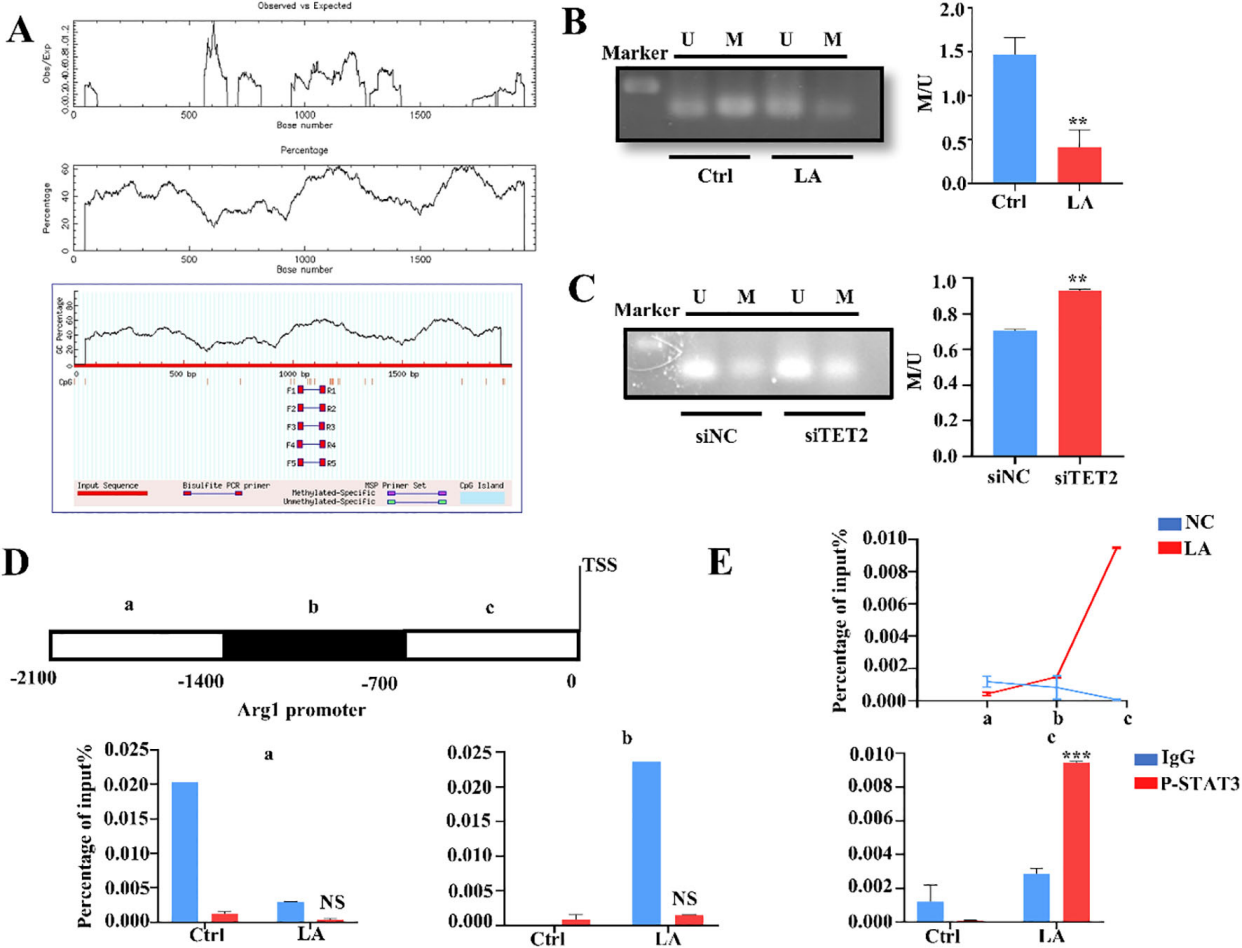
TET2 regulates the methylation levels of the Arg1 promoter region in MDSCs. **(A)** Utilize online databases to predict methylation sites within the Arg1 promoter. **(B)** Methylation-specific PCR was performed to evaluate changes in methylation levels within the Arg1 promoter region following lactate treatment. **(C)** Methylation-specific PCR was performed to evaluate changes in methylation levels within the Arg1 promoter region following siTET2 treatment. **(D)** Design of TET2 promoter primers of Arg1. **(E)** ChIP-qPCR assay of TET2 status in the Arg1 genomic region in MDSCs. All quantitative data represent the mean ± SD of n = 6 independent biological replicates. The findings were consistent across three independent experimental replicates. **p < 0.01, ***p < 0.005; NS, no significance.

We searched UCSC database (https://genome.ucsc.edu) for the Arg1 promoter sequence and predicted methylation sites using MethPrimer (https://www.urogene.org/methprimer). Results revealed multiple methylation sites near the Arg1 transcription start site (**Figure 5A**). MSP-PCR examined methylation level changes in the Arg1 promoter region after lactate treatment and after TET2 knockdown (**Figures 5B, C**). Methylation levels significantly decreased in the Arg1 promoter region after lactate treatment and increased noticeably after TET2 knockdown. Finally, ChIP-qPCR confirmed TET2 enrichment in the Arg1 promoter region (**Figures 5D, E**). The underlying mechanism involves TET2 acting as a demethylase that targets methylated regions within the ARG1 promoter, thereby upregulating its expression.

### p-STAT3 and TET2 interact to jointly regulate the methylation levels of the Arg1 promoter region

**Figure 6:**
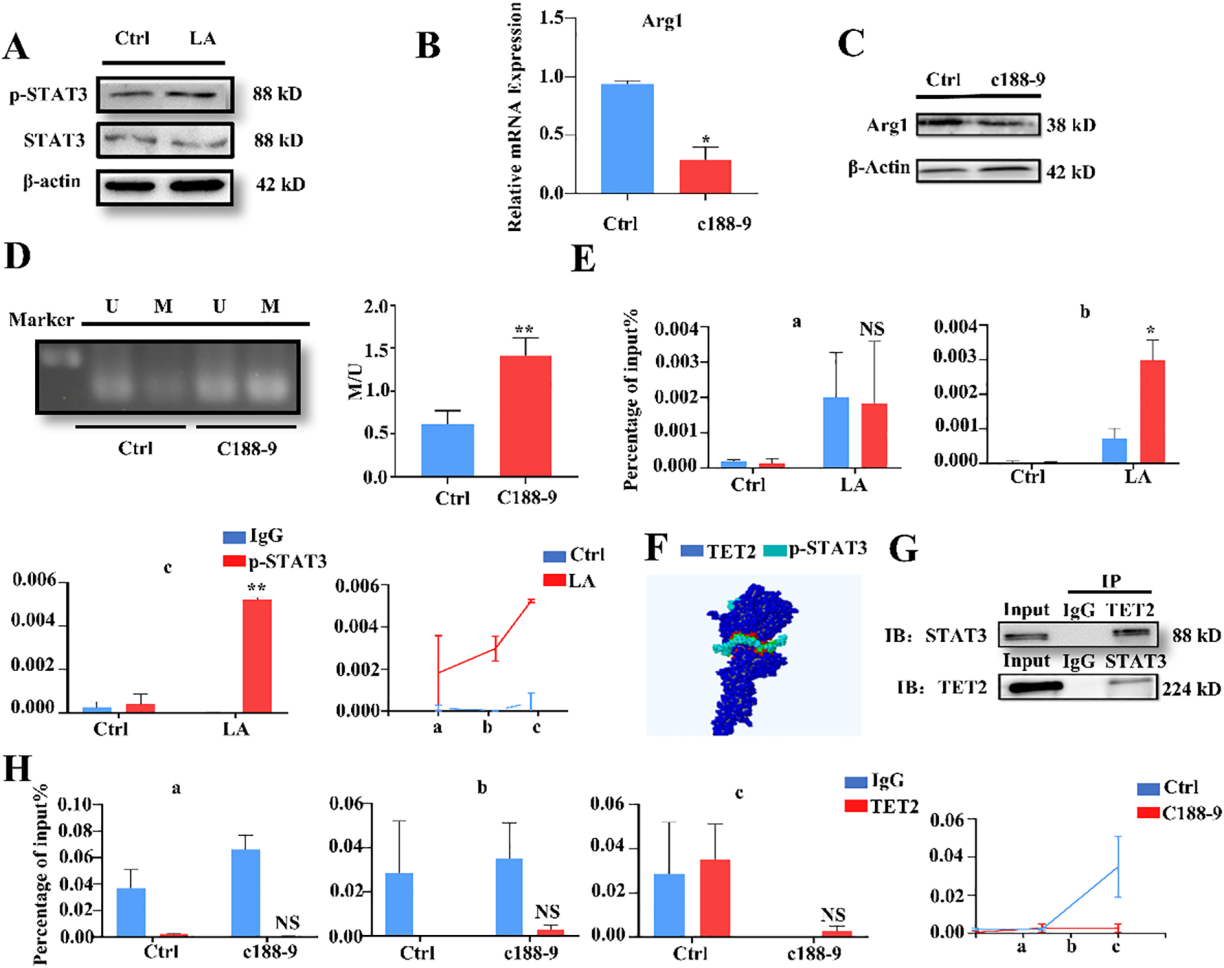
p-STAT3 and TET2 interact to jointly regulate the methylation levels of the Arg1 promoter region. **(A)** Western blotting analysis was conducted to evaluate changes in STAT3 phosphorylation levels following lactate treatment. **(B)** qRT-PCR analysis was conducted to evaluate the expression levels of Arg1 in MDSCs following treatment with C188-9. **(C)** Western blotting analysis was conducted to evaluate the expression levels of Arg1 in MDSCs following treatment with C188-9. **(D)** Methylation-specific PCR analysis was conducted to assess the methylation status of the Arg1 promoter region following C188–9 treatment. **(E)** The binding of p-STAT3 to the Arg1 promoter region was determined using ChIP after C188–9 treatment. **(F)** The Z-dock database was utilized to predict the binding affinity and interface of TET2 and STAT3. **(G)** Co-immunoprecipitation (Co-IP) analysis was conducted to evaluate the interaction between TET2 and p-STAT3. **(H)** ChIP analysis was conducted to evaluate TET2 binding to the Arg1 promoter region after C188–9 treatment. All quantitative data represent the mean ± SD of n = 6 independent biological replicates. The findings were consistent across three independent experimental replicates. *p < 0.05, **p < 0.01; NS, no significance.

Lacking a DNA-binding domain, the DNA demethylase TET2 requires cofactors to function (30). Research indicates STAT3 not only acts as a signaling molecule but also as a cofactor enhancing TET2 function (31, 32). Therefore, we investigated whether STAT3 assist TET2 in regulating Arg1 promoter methylation. After lactate treatment, STAT3 phosphorylation (p-STAT3) was significantly upregulated (**Figure 6A**). Treatment with the STAT3 phosphorylation inhibitor C188-9 (20 mM) decreased Arg1 mRNA and protein levels (**Figures 6B, C**). Inhibition of STAT3 phosphorylation also resulted in a significant increase in Arg1 promoter methylation (**Figure 6D**), suggesting p-STAT3 leads to decreased methylation of the Arg1 promoter, upregulating its expression. ChIP-qPCR demonstrated that p-STAT3 enrichment in the Arg1 promoter region (**Figure 6E**) indicating its involvement in regulating Arg1 promoter methylation.

To further investigate the relationship between p-STAT3 and TET2, we predicted potential interaction using the Z-dock database (https://zdock.umassmed.edu). Results showed a distinct binding interface between TET2 (blue) and STAT3 (green) (**Figure 6F**). Co-immunoprecipitation confirmed the interaction between TET2 and p-STAT3 (**Figure 6G**). Finally, ChIP-qPCR after treating MDSCs with C188–9 showed decreased enrichment of TET2 in the Arg1 promoter region (**Figure 6H**). These results suggest that p-STAT3 and TET2 interact and jointly regulate Arg1 promoter methylation levels.

## Discussion

Due to the Warburg effect, the TME is characterized by high lactate levels, promoting tumor progression and immune evasion through various mechanisms (33). Histone lactylation, a recently discovered epigenetic modification (12), warrants further investigation in immune cells. Previous studies from our group showed lactate regulates MDSCs and tumor cells. This study further investigated lactate regulation of MDSCs. We found lactate significantly upregulates MDSCs immunosuppressive function. High lactate levels increase lactylation, upregulating TET2 expression. TET2 subsequently demethylates the promoter region of ARG1, leading to the upregulation of its expression in MDSCs. Our data reveal a novel mechanism: lactate regulates TET2 in MDSCs, affecting downstream key genes.

**Figure 7:**
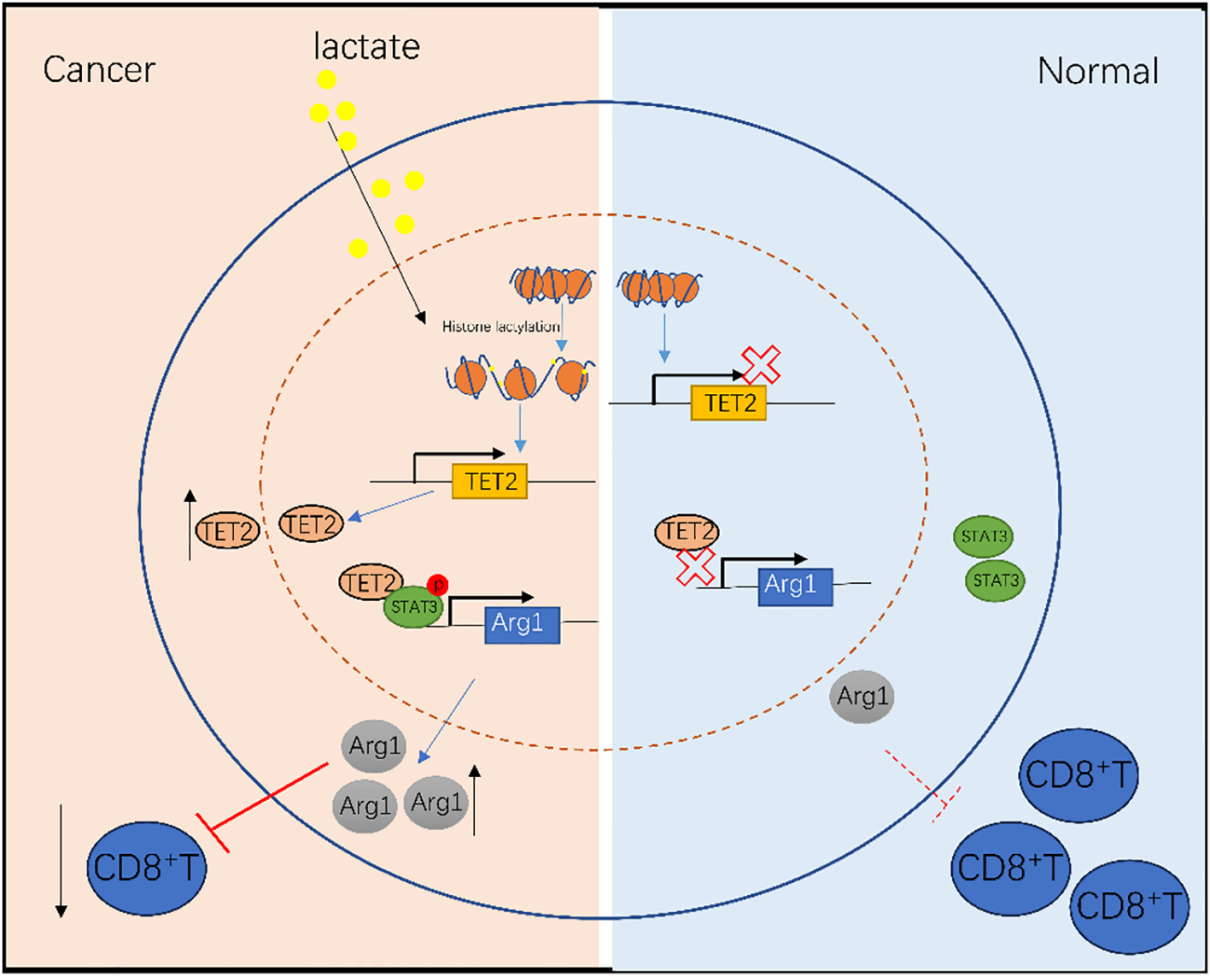
Schematic of the immunosuppressive lactate–TET2–Arg1 pathway in MDSCs.

TETs are crucial demethylases, playing significant roles in maintaining methylation stability (34). Among them, TET2 lacks a DNA-binding domain, relying on auxiliary factors for DNA binding. Studies show TET2 binds transcription factors like SNIP1 and HNF4α to perform demethylation (30, 35). Arg1 is a key immunosuppressive molecule in MDSCs. Its secretion depletes arginine in the TME, linked to nitric oxide consumption and T cell dysfunction (36). STAT3 is a crucial regulator of MDSCs and a recognized transcription factor for Arg1 (37). Our research connects these molecules. We first verified the STAT3-TET2 interaction bioinformatically and by CO-IP. Subsequently, inhibiting STAT3 phosphorylation significantly decreased TET2 binding to the Arg1 promoter, indicating p-STAT3 regulates TET2 binding to Arg1. These findings enrich the understanding of TET2 regulation in MDSCs, expand the role of p-STAT3 in regulating Arg1, and provide new targets for clinical tumor therapy (**Figure 7**).

Building on our findings, hyperlactate-induced lactylation significantly promotes tumor development, highlighting the promise of lactylation inhibitors. Current strategies to suppress lactylation include reducing lactate production via glycolytic inhibitors (e.g., 2-DG) or LDHA/B siRNA, inhibiting the lactylation writer p300, or enhancing delactylases such as HDAC1 and SIRTs. This study newly identifies a lactylation–TET2–STAT3 axis, suggesting that targeting any component may disrupt lactylation. However, as STAT3 acts as a pleiotropic transcription factor, its inhibition may lack specificity and require further validation for precise therapeutic intervention.

Beyond its role identified in our study as a cofactor facilitating TET2-mediated demethylation within the lactate-driven pathway, STAT3 itself can be functionally modulated by lactylation, given its identity as a pivotal transcription factor. It is plausible that lactylation could also occur on STAT3, given the reported acetylation of STAT3 by p300. Such lactylation may alter STAT3’s activity and impact downstream gene expression (38). This mechanism, while highly relevant to our findings, represents a distinct regulatory axis: in our work, STAT3 acts as a bridging partner, whereas its direct lactylation modifies its core function. The capacity of STAT3 to engage with lactylation through multiple modes underscores that a single molecule can be regulated in diverse ways, significantly broadening the perspective for future research in this field.

Our study is subject to several limitations. Firstly, our analysis relied on MDSCs isolated from the spleen. Since the properties of splenic MDSCs can differ from those of tumor-infiltrating MDSCs (T-MDSCs), our conclusions may not directly translate to the tumor microenvironment and need confirmation in T-MDSCs. Secondly, beyond the genes identified here, the full spectrum of targets co-regulated by TET2 in MDSCs remains an open question. Lastly, the mechanistic details of how STAT3 facilitates TET2 function await further elucidation.

In summary, this study integrates metabolism, epigenetics, and tumor immunology to elucidate how TET2 regulates MDSCs immunosuppression. It identifies specific lactate-regulated sites on TET2 and demonstrates how TET2 regulates Arg1. These findings provide new insights into lactate regulation of MDSCs and guidance for identifying novel anti-tumor targets. Future research will further explore TET2’s role in the TME and investigate regulatory networks of other key genes, contributing to advances in anti-tumor treatment.

## Conclusion

This study investigated the regulation of TET2 by histone lactylation in MDSCs. The elucidated mechanism is that lactate-mediated histone lactylation upregulates TET2 expression. TET2, a DNA demethylase, subsequently demethylates target genes like Arg1. Given TET2’s role as a crucial tumor suppressor and its importance in immune cells, these findings suggest its potential as a novel immunotherapeutic target and biomarker.

## Data Availability

The original contributions presented in the study are included in the article/**Supplementary Material**. Further inquiries can be directed to the corresponding authors.

## Supplementary material

Supplementary Figure 1 Purity plot of MDSCs and CD8 ^+^T cells. **(A)** Flow cytometry revealed the purity of MDSCs. **(B)** Flow cytometry revealed the purity of CD8 ^+^T cells.
