# Supplementary figures and images for: Histone lactylation-derived TET2 enhanced Arg1-mediated MDSC immunosuppression
Source: Front Immunol. 2026 Jan 16;16:1677780. doi: 10.3389/fimmu.2025.1677780 (PMC12855476; doi:10.3389/fimmu.2025.1677780)

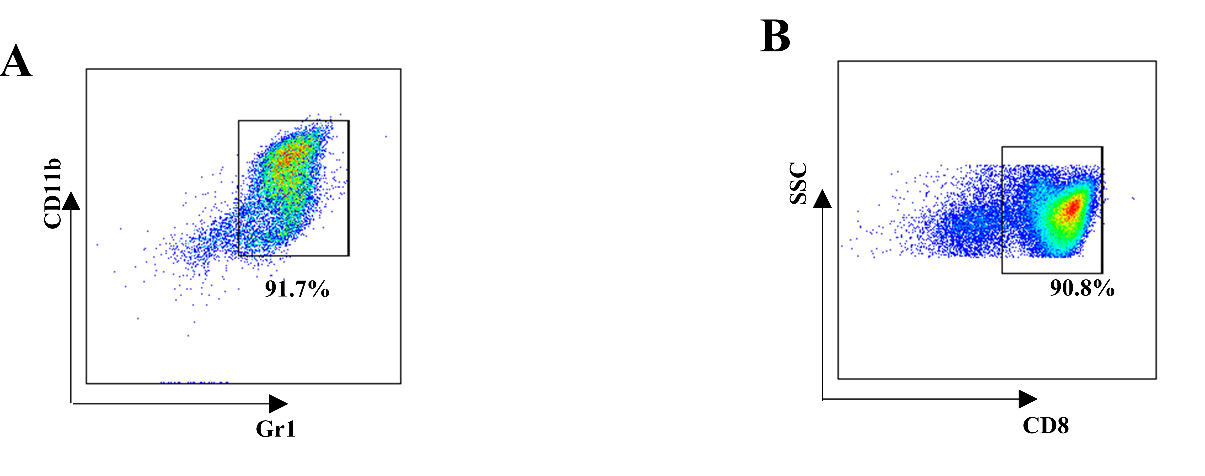

Supplement: Supplementary Figure 1 — Purity plot of MDSCs and CD8 +T cells. (A) Flow cytometry revealed the purity of MDSCs. (B) Flow cytometry revealed the purity of CD8 +T cells. [file Image1.tif]
